# Better climate action through the right knowledge? Development and validation of an item-response-theory scale measuring climate effectiveness knowledge

**DOI:** 10.3389/fpsyg.2024.1347407

**Published:** 2024-11-14

**Authors:** Clara Elisa Simon, Martin Julian Merten

**Affiliations:** Department of Environmental Psychology, Institute of Psychology, Otto von Guericke University Magdeburg, Magdeburg, Germany

**Keywords:** environmental behavior, environmental knowledge, environmental impact, effectiveness knowledge, scale development, measurement, partial credit model, climate action

## Abstract

Knowledge about the relative environmental impact and climate-protective potential of different actions (*effectiveness knowledge*) is important for successful sustainable action. However, there is currently no scale for measuring effectiveness knowledge that meets psychometric quality criteria. We developed a new scale consisting of 16 ranking and choice tasks and tested it on a convenience sample of 278 people from Germany in an online study. The final scale version achieved a reliability of *rel* = 0.655. This is significantly higher than the reliability of 0.329 achieved by an established knowledge scale used for comparison. Inter-correlation of both scales was moderate to strong, but the new scale is able to explain 3% additional variance in high-impact pro-environmental behavior when controlling for environmental attitude, whereas the established scale is not explaining any additional variance, indicating incremental validity of our scale. We conclude that it is possible to use ranking tasks to measure effectiveness knowledge more reliably in a test-efficient way and provide a set of items which are usable in the contemporary German context.

## Introduction

Anthropogenic climate change is one of the greatest and most urgent challenges of our time (Steffen et al., 2015; World Economic Forum, 2022, 2023). In order to achieve the goal of the Paris Climate Agreement to limit global warming to well below 2°C, preferably 1.5°C, a drastic reduction of greenhouse gas emissions is necessary in the upcoming years [German Advisory Council on the Environment (Sachverständigenrat für Umweltfragen), 2020; United Nations, 2015]. Over 60% of global greenhouse gas emissions can be directly or indirectly linked to private households, with mobility, housing and food being the most important consumption categories causing emissions (Ivanova et al., 2016). Therefore, emission reductions on the household level play an important role in tackling climate change: living car-free, flying less, using renewable electricity and switching to a vegan diet are some behavioral changes with a particularly large impact in the respective categories (Ivanova et al., 2020). Adopting these behavioral changes means using fewer fossil fuels, resulting in less greenhouse gas emissions (for mobility and heating; Ivanova et al., 2020) or, among other emission sources, less methane emissions and land use change for feed production (for vegan diet; Poore and Nemecek, 2018). In order to make meaningful behavior changes, people need to know about their options for action (Cologna et al., 2022). Knowledge about the relative efficiency of different behavioral options is necessary and useful: no one has infinite resources for action, so identifying and starting at the “big points” with a particularly large climate impact is a good strategy (Bilharz, 2004; Cologna et al., 2022; Nielsen et al., 2021a). Evidence shows, however, that this so-called “effectiveness knowledge” (Frick, 2003; Kaiser and Fuhrer, 2003) is insufficient in most people. For example, the climate impact of avoiding plastic or adopting a regional-seasonal diet tends to be overestimated, while the impact of a vegetarian or vegan diet is underestimated (Cologna et al., 2022; de Boer et al., 2016; Tofighi and Jackson, 2020; Wynes et al., 2020). Curtailment actions tend to be overestimated in their impact, while efficiency improvements tend to be underestimated (Attari et al., 2010; Gardner and Stern, 2008). The self-assessed sustainability of one's lifestyle correlates only very weakly with the actual ecological footprint (Bleys et al., 2018). However, to measure the effects of knowledge interventions and get a better understanding of the relationship between effectiveness knowledge and high impact pro-environmental behavior, it is necessary to have a reliable scale.

## Theoretical background

The knowledge that allows us to estimate the efficiency of possible environmental actions and thus make cost-benefit considerations, selecting particularly efficient actions from many alternatives, is referred to as effectiveness knowledge in the scientific literature (Frick, 2003; Kaiser and Fuhrer, 2003). It is one of three types of environmental knowledge postulated by Frick (2003), besides system knowledge (knowledge about causalities in ecosystems, e.g. how climate change occurs) and action knowledge (knowledge about possible courses of action and their implementation). It is assumed that effectiveness knowledge builds on system and action knowledge and influences environmental behavior (Frick, 2003; Frick et al., 2004; Roczen et al., 2014).

In this study, the focus will be on effectiveness knowledge concerning greenhouse gas emissions, due to their particular relevance for anthropogenic climate change (Steffen et al., 2015), although according to Frick (2003), it can also be applied to other areas of environmental protection.

### How effectiveness knowledge and behavior are connected

Previous findings on effectiveness knowledge are rather mixed: Frick (2003) and Frick et al. (2004) found a moderate correlation of *r* = 0.29 between effectiveness knowledge and general environmental behavior in their large-scale study. Roczen et al. (2014) found no significant influence of effectiveness knowledge on general environmental behavior in a student sample. Braun and Dierkes (2019) also found only very weak correlations between effectiveness knowledge and environmental behavior intentions (*r* = −0.06, *r* = 0.08, and *r* = 0.10, respectively, at the three measurement times) in a knowledge intervention for students. In a study by de Almeida Barbosa et al. (2021), effectiveness knowledge levels did not differ between climate activists and non-activists. Cologna et al. (2022), reported a moderate correlation between the competence to correctly assess the effectiveness of different action options on a Likert scale and the willingness to perform high-impact actions (*r* = 0.20).

In the following, however, we argue that the rather weak relationship between effectiveness knowledge and environmental behavior may be mainly due to three methodological problems regarding the measurement of knowledge and behavior as well as the influence of environmental attitude.

### Problem 1: Measuring effectiveness knowledge

We know of four studies that designed effectiveness knowledge measures using IRT modeling (Díaz-Siefer et al., 2015; Frick, 2003; Geiger et al., 2014; Roczen et al., 2014). These studies have made important contributions in distinguishing the three types of environmental knowledge and making them measurable; however, the effectiveness knowledge subscales showed poor measurement properties – especially in form of low reliabilities, e.g. *rel*_*P*_ = 0.50 (Frick, 2003) and *rel*_*P*_ = 0.45 (Díaz-Siefer et al., 2015) or unclear reliability estimates (Geiger et al., 2014; Roczen et al., 2014). Moreover, all four effectiveness knowledge scales were too difficult. Accordingly, the variance on the construct effectiveness knowledge was low. This floor effect likely reduced the correlation with environmental behavior (Roczen et al., 2014). The high difficulty could be related to the way the items are designed: the four scales measuring effectiveness knowledge often ask for specific numbers [e.g., “*How much less do LED bulbs spend compared to conventional bulbs? (a) 30%, (b) 45%, (c) 70%, (d) 80%, (e) 100%”* from Díaz-Siefer et al., 2015], and the content asked is sometimes quite far from everyday decisions and these scales are now partly outdated. They are also not designed with a primary content focus on climate impacts but address a broader range of topics with questions on energy, water, and other resource use.

Some newer studies do not use validated scales at all, but *ad-hoc*-measures (Cologna et al., 2022; Tofighi and Jackson, 2020; Wynes et al., 2020) to identify the effectiveness knowledge gap. Some indirectly inferred a possible knowledge gap from the weak relationship between self-assessed sustainable lifestyle and actual footprint (Bleys et al., 2018; Gatersleben et al., 2002; Moser and Kleinhückelkotten, 2018).

### Problem 2: Which kind of pro-environmental behavior?

A successful use of effectiveness knowledge would be to behave in a climate-friendly manner in areas with a high impact. However, most studies only investigated general ecological everyday behavior in different domains as a dependent variable (Frick, 2003; Frick et al., 2004; Geiger et al., 2014; Roczen et al., 2014) using the General Ecological Behavior Scale, which does not take impact into account (Kaiser and Wilson, 2004). Thus, the low correlation could partly be due to the choice of the dependent variable. Evidence for this idea can be found in Cologna et al. (2022): in their study, effectiveness knowledge was a positive predictor of intentions to perform high-impact behaviors, but a weakly negative predictor of intentions to perform low-impact behaviors. Accordingly, a more appropriate criterion for the effects of effectiveness knowledge would be pro-environmental behavior with high climate impact (high-impact PEB). Current contributions to the environmental psychological research discourse also call for a focus on impact in order to make a relevant contribution to mitigating climate change (Kennedy et al., 2015; Nielsen et al., 2021a,b).

### Problem 3: The role of environmental attitude in the knowledge-behavior-connection

Effectiveness knowledge is unlikely to lead to action equally for everyone: since good knowledge about the climate effects of one's actions should only lead to behavior change if one actually cares about protecting the climate, the effects of knowledge are likely to be moderated by environmental attitude. Some evidence on this assumption can also be found in the literature: for example, two studies of smart meter feedback interventions for energy conservation (which provide knowledge about the effectiveness of one's actions) consistently concluded that the intervention was effective only for individuals with a high environmental attitude (Henn et al., 2019; Puntiroli and Bezençon, 2020). Furthermore, it is generally assumed that high environmental attitude fosters the acquisition of new environmental knowledge. Such a relationship was shown for example in a cross-sectional correlational study by Attari et al. (2010), as well as in more recent experimental studies (e.g. Baierl et al., 2022). As literature provides evidence that environmental attitude is strongly related to climate-friendly behavior, including some high-impact domains (Bruderer Enzler and Diekmann, 2019; Gatersleben et al., 2002; Kennedy et al., 2015), this makes it harder to distinguish the effects on behavior of environmental attitude vs. effectiveness knowledge. The question whether effectiveness knowledge explains additional variance in high-impact behavior (and to what extent), when the problems mentioned above are addressed, remains open.

## Aims of this study

The objective of this study is to develop and evaluate an improved test to measure (objective) climate effectiveness knowledge by addressing the aforementioned problems in the scale construction and the validation design. Content-wise, the scale will be limited to only one evaluation dimension, namely the efficient avoidance of global warming potential. This makes it possible to objectively specify one correct order, as the impacts of different greenhouse gases as well as contributors to climate change, that are not entirely based on greenhouse gas emissions, like land-use change and air-travel related changes in the upper atmosphere, can be measured in the common unit of CO_2_-equivalents. Adding further dimensions (e.g. biodiversity loss, pollution, water usage), albeit interesting, would require these dimensions to be offset against each other. There is no scientific consensus on how this could be done, e.g. how much biodiversity loss equals how many CO_2_-equivalents. We chose the dimension of global warming potential because climate change is (alongside biodiversity loss), the most urgent and important planetary boundary and in turn influences all other planetary boundaries (Steffen et al., 2015). Additionally, global warming potential is easier to quantify than biodiversity loss.

The scale was constructed with the goal of being closer to everyday life and less difficult in comparison with previous scales for assessing effectiveness knowledge (Díaz-Siefer et al., 2015; Frick, 2003; Frick et al., 2004; Roczen et al., 2014). This is to be achieved using ranking tasks. These have the advantage that the probability of finding the absolute correct solution by guessing is low. Furthermore, since ranking tasks with four items contain six pairwise comparisons, significantly fewer items are needed to achieve the same test accuracy compared to single-choice tasks. The number of items in existing effectiveness knowledge scales (Díaz-Siefer et al., 2015; Frick, 2003; Roczen et al., 2014) varies between 20 to 30 single-choice items with two to five response options each, with low reliabilities. Our scale should achieve higher reliabilities than the established scales with a similar number of items, aiming for a reliability of at least 0.70 (Moosbrugger and Kelava, 2012).

H1: The reliability of our newly developed effectiveness knowledge scale is higher than the reliability of an established scale.

### Construct validity

The test scores of the new scale should show a high correlation with the test scores of an established effectiveness knowledge scale, as both measure the same construct (Schmidt-Atzert and Amelang, 2012). Given that existing effectiveness knowledge scales cover a broader range of topics than our new one, which is only about factors contributing to climate change, and the fact that floor effects were found in past measurements, limiting variance and thus correlations with other constructs (Roczen et al., 2014), a moderate correlation would also be a satisfactory indication of convergent validity.

A positive relationship is also expected between effectiveness knowledge and educational level, as it seems reasonable that individuals with a higher level of education have acquired more knowledge about climate change (Kollmuss and Agyeman, 2002). For example, in the study by Díaz-Siefer et al. (2015), the correlation between environmental knowledge and educational level was *r* = 0.46. In the study by Cologna et al. (2022) et al. this correlation (measured with *ad-hoc* scales, see above) was smaller, at *r* = 0.17. We therefore put forward the following hypotheses:

H2: There is at least a moderate positive relationship between test scores on the newly developed effectiveness knowledge scale and test scores on an established effectiveness knowledge scale.

H3: There is a moderate positive relationship between effectiveness knowledge and educational level.

### Criterion validity

Another goal is to test whether the new scale for effectiveness knowledge can predict high-impact environmental behavior. Since knowledge is a distal predictor of behavior, and in line with previous studies, no large relationship is expected.

H4: There is a weak to moderate positive relationship between effectiveness knowledge and high-impact PEB.

H5: This relationship is stronger for effectiveness knowledge measured with the new scale than for effectiveness knowledge measured with an established scale.

This relationship should be particularly evident in groups with high environmental attitude, as motivation to protect the environment should help translate knowledge into action, while knowledge could help translate an existing motivation into effective action. Effectiveness knowledge, however, should also play a significant role in explaining behavioral variance beyond environmental attitude in order to be a construct of interest. The usefulness of the newly constructed knowledge scale should also be shown in comparison to an established effectiveness knowledge scale. Only then would the new scale represent a practically relevant improvement. This leads to the following four hypotheses:

H6: Environmental attitude has a moderating influence on the knowledge-behavior relationship: the higher the environmental attitude, the stronger the influence of effectiveness knowledge on high-impact PEB.

H7: There is a moderate positive relationship between effectiveness knowledge and environmental attitude.

H8: Effectiveness knowledge makes a significant contribution beyond environmental attitude in explaining high-impact PEB.

H9: This incremental contribution is larger for effectiveness knowledge measured with the new scale than for effectiveness knowledge measured with an established scale.

## Methods

### Instruments

#### Developing a new effectiveness knowledge scale

The first step of scale construction was an extensive research on the global warming potential of different products, services and actions, in the areas of food, mobility, electricity, heating and other private consumption. The research was primarily based on current literature from Germany that included the contributions to climate change of the most prominent greenhouse gases, as well as land-use change and non-carbon related changes in the upper atmosphere caused by air-travel, if applicable. Based on this data, 13 ranking tasks and 13 single-choice tasks were constructed (see Supplementary Appendix A1 for the items and the sources of the corresponding CO_2_-equivalents). The ranking tasks each required four elements to be placed in the correct order, while the single-choice tasks involved either “How much of X equals Y?” type questions or finding the most climate-friendly/harmful among several alternatives. Two pretests with *N* = 5 and *N* = 2 were conducted to identify and reword items that were misleading or too difficult.

#### Item-response-theory

The existing scales measuring effectiveness knowledge by Frick et al. (2004), Roczen et al. (2014), Geiger et al. (2014), and Díaz-Siefer et al. (2015) were constructed based on the Rasch model (RM; Rasch, 1960/1980). The RM attempts to explain response patterns by only two parameters: Person ability θ_*i*_ and item difficulty β_*j*_. Both are mapped onto a common dimension (Bond and Fox, 2007; Strobl, 2015) using the model equation


$$\begin{array}{l} P\left(U_{ij}\;=\;1|\theta_{i},\beta_{j}\right)\;=\;\frac{e^{\theta_{i}-\beta_{j}}}{1+e^{\theta_{i}-\beta_{j}}} \end{array}$$

Strobl (2015), which describes a logistic function. The higher the person ability is compared to the item difficulty, the higher the probability of solving the item. When ability and difficulty are equal, the solving probability is 50%. The items in an ideal Rasch-homogeneous test only differ from each other in terms of difficulty, so there is only one latent dimension.

Measurements based on the Rasch model have several advantages over measurements based on classical test theory (CTT): firstly, the Rasch model's validity can be empirically tested. Thus, it can be determined whether it is justified to build a person sum score over the items, whereas in the CTT this is mostly done untested (Moosbrugger, 2012; Wright and Masters, 1982). Secondly, for CTT-scales, all items must be of approximately similar, moderate difficulty, whereas items in the RM can be spread over a broad range of difficulty and measure accurately even at the extremes (Bond and Fox, 2007). Additionally, individuals can be compared in terms of ability even if they do not answer the identical item set, provided some items are present in both sets and all items are from a Rasch-homogeneous item pool (Sälzer, 2016). Because of these advantages, the RM is becoming increasingly popular, especially in performance testing. Well-known examples of its use include international educational studies such as PISA or TIMSS (Sälzer, 2016).

One disadvantage of the RM is that it only knows correct or incorrect answers. Therefore, it is not suited for tasks in which partially correct answers are possible. For this purpose, there is an extension of the Rasch model, for which the properties and advantages explained above also apply: the partial credit model (Masters, 1982). The formula for this model is:


$$P\left(U_{ij}\;=\;c\text{ |}\theta_{i},\delta_{j1},\ldots .\delta_{jm_{j}}\right)\;=\;\frac{e^{\sum_{k\;=\;0}^{c}\left(\theta_{i}-\delta_{jk}\right)}}{\sum_{l\;=\;0}^{m_{j}}e^{\sum_{k\;=\;0}^{l}\left(\theta_{i}-\delta_{jk}\right)}}$$

The formula considers the probability of the response falling into category c, where c may be any value between 0 and *m*_*j*_. This probability depends on the person ability θ_*i*_, and the threshold parameters δ_*j*1_ to δ_*j*_*m*__*j*__. Thus, the difference to the Rasch model is that an item has not only a single difficulty β_*j*_, but multiple difficulty thresholds δ_*jk*_.

As our approach for developing the new scale includes ranking tasks, which by their nature can be partially correct, the partial credit Rasch model seemed the most appropriate model for the construction and statistical analysis of our scale.

#### Selection of other scales

##### Established effectiveness knowledge scale

We used the scale developed by Roczen et al. (2014), which is based on the RM and consists of 29 single-choice items. Three of them are very similar to three items from the newly developed scale, so they were only asked once and treated as though they had occurred in both scales.

The scale was analyzed based on the dichotomous RM. The mean item difficulty was set to zero (sum normalization). Missing values rarely occurred (0.2% across all items on average) and were treated as incorrect answers. There were no participants or items with a perfect or zero score. The mean value of the person parameters was *M* = 0.57 (*SD* = 0.55). The scale showed a very low person-separation reliability of *rel*_*P*_ = 0.329. Three items showed signs of underfit.

##### Environmental attitude

We used the subscales on environmental affect (seven items) and environmental cognition (eight items) from the 2018 German national environmental attitude survey (Rubik et al., 2019). We followed Geigers suggestion, that these form a unidimensional construct due to a latent model correlation of *r* = 0.97. A detailed presentation of the scale construction can be found in Geiger (2020). Two items were slightly modified in wording from the original to increase comprehensibility. Unlike in the environmental attitude survey, where the items were assessed on a four-point Likert scale, we used a five-point Likert scale with an additional “cannot judge”-option.

Scores were coded with one (low environmental attitude) to five (high environmental attitude). Across all items, there were on average 1.4% missing values. If missing values occurred, the mean was calculated only over the existing values. The scales mean is *M* = 4.35 (SD = 0.48). The distribution is left-skewed (*skewness* = −1.18, Shapiro–Wilk test: *W* = 0.92, *p* < 0.01). The scale shows a satisfactory reliability (α = 0.86), with one item having a discriminatory power *r* < 0.30.

##### High-impact pro-environmental behavior (high-impact PEB)

We compiled a 17-item *ad-hoc* scale from several sources (KlimAktiv gemeinnützige Gesellschaft zur Förderung des Klimaschutzes mbH, 2020; Kaiser and Wilson, 2004; Bruckmann, 2020). The items cover different areas of high-impact private consumption (electricity, heat, mobility, and food), but also political behavior such as participation in political demonstrations and donations to climate and environmental causes. The scale consists of ten five-point frequency Likert items (“never”/“rarely”/“occasionally”/“often”/“very often or always”/“no answer”), three dichotomous items (“yes”/“no”) and four items with an individual response format, in which, for example, the number of hours flown in the last year are to be entered. All items were coded with values from zero to four, where zero corresponds to climate-harmful behavior and four to climate-friendly behavior. The item regarding high-sea cruises was excluded because it was negated by all subjects. The items and their coding details can be found in Supplementary Appendix A2.

The score for each person is the mean value across the 16 items. Across all items, there were on average 4.9% missing values. If missing values occurred, the mean was calculated only over the existing values. The scale shows a reliability of Cronbach's α = 0.69, with a mean of *M* = 2.21 (SD = 0.53). Seven items show a low item-scale correlation of *r* < 0.30. One item (acquisition of a solar system) is even negatively correlated with the overall scale. Nevertheless, all items were retained since the objective was to map the climate impact of the individuals as well as possible.

### Participants and procedure

The survey was conducted online in the period from December 2021 to February 2022 via the platform *SoSci-Survey* (Leiner, 2022). The study could be completed on a desktop computer, smartphone, or tablet. The questionnaire took an average of 24.83 minutes to complete (SD = 5.81).

The order of the scales was as follows: (1) new effectiveness knowledge scale, (2) established knowledge scale (both framed as quizzes, with the instruction to answer by the best of one's knowledge without researching), (3) question on which quiz version the participant preferred and why, (4) environmental attitude scale, (5) high-impact PEB scale, (6) feedback. In the feedback section, participants could make general comments on the questionnaire and download the solutions for the two knowledge scales. In addition, an individual knowledge score was returned to everyone. Within each scale, the order of items was randomized. Two attention checks were randomly placed within the knowledge scales.

The preliminary target sample size was ~200, which according to the power analysis was sufficient to reveal an incremental variance explanation of 5% in a regression model with three predictors at a power of 90% (Faul et al., 2007). Participants were mainly recruited in the private environment of the first author as well as via mailing lists and postings in social media. Furthermore, about 70 students of the bachelor's program in psychology of our university participated, in exchange for credit points.

#### Characteristics of the sample

The questionnaire was started 360 times. Of these, 278 tests (77%) were completed. These *N* = 278 cases were used for the item analyses of the new effectiveness knowledge scale. For testing the validation hypotheses, one subject was excluded due to a very low completion time and one was excluded due to too many missing values on the environmental attitude and environmental behavior scales, resulting in a sample of *N* = 276.

Of these 276 subjects, 60% were female, 38% male and 1% diverse. The mean age of the sample was 32.43 years (SD = 14.34) with a median of 26 years, and a range from 16 to 79 years. The educational level of the sample was very high (see Table 1). More than 50% of the participants had a university degree, and only about 10% did not have qualifications for higher education. Of the 223 people who reported their household income (see Table 1), a mean of 2.25 people lived in the household (SD = 1.13, median = 2).

**Table 1:** Educational level and income of the sample.

**Educational level**	**Absolute**	**In percent**
**Low (no qualification for higher education)**		
Still at school	4	1.45%
Secondary school certificate (*Realschulabschluss* or equivalent)	6	2.17%
Completed professional training/apprenticeship	18	6.52%
**Medium (qualified for higher education)**		
Vocational baccalaureate, advanced technical college entrance qualification	7	2.54%
High school diploma, university entrance qualification	99	35.87%
**High (university degree)**		
Bachelor	61	22.10%
Master or equivalent (*Diplom, Magister* or *Staatsexamen*)	69	25.00%
PhD	12	4.35%
**Monthly net household income**		
< 1.000 €	50	18.12%
1.000 € to < 2.000€	51	18.58%
2.000 € to < 3.000€	42	15.22%
3.000 € to < 4.500€	42	15.22%
4.500 € to < 6.000€	26	9.42%
More than 6.000€	12	4.35%
Not stated	53	19.20%

### Analysis of the new effectiveness knowledge scale

#### Software and scoring

All statistical analyses were performed using R (R Core Team, 2021) in RStudio (RStudio Team, 2022). The analysis of the newly developed effectiveness knowledge scale was based on the partial credit model (Masters, 1982) using the R package eRM (Mair et al., 2021).

**Figure 1:**
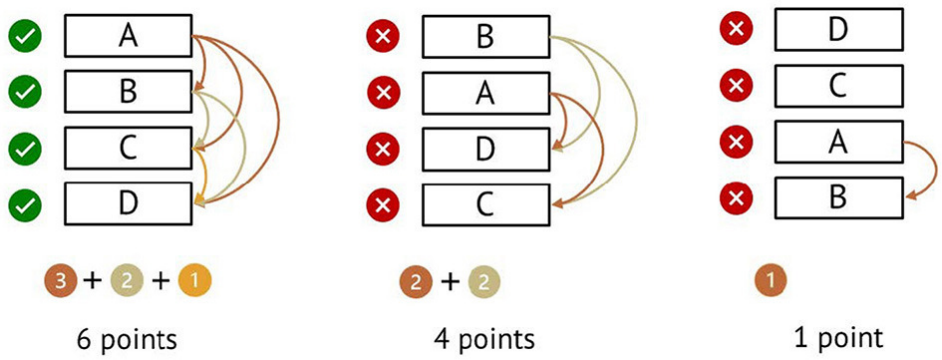
Example scores with a correct arrangement of ABCD. The arrows mark the correct pair comparisons, so the number of arrows corresponds to the number of points.

For scoring the ranking tasks, we checked for all six possible pair comparisons whether they were solved correctly. One point was awarded for each correct pair comparison, corresponding to a score between 0 and 6 points for each ranking task (see Figure 1). Single-choice tasks were coded with 1 (correct) or 0 (incorrect). Missing values rarely occurred (0.3 % across all items on average) and were treated as incorrect responses.

#### Category merging

As can be seen in Table 2, most people scored at least three points on most of the ranking tasks. Some score categories remained empty or covered by only a few people. Empty categories do not contain any information, so parameter estimation is impossible for these. Therefore, categories were combined: starting at zero points, for each item, each point category with fewer than 20 people was merged with the next higher category until there were at least 20 people in each merged category.

**Table 2:** Frequency distribution of points per item.

**Ranking tasks**								**Single-choice-tasks**		
**Item**	**0 P**.	**1 P**.	**2 P**.	**3 P**.	**4 P**.	**5 P**.	**6 P**.	**Item**	**0 P**.	**1 P**.
EK01	*2*	*8*	*8*	31	69	97	63	EK13	*8*	270
EK02	*4*	*8*	*15*	28	78	81	64	EK14	217	61
EK03	*14*	*2*	***0***	*4*	*12*	35	211	EK15	171	107
EK04	*3*	*2*	24	47	67	92	43	EK16	116	162
EK05	***0***	*2*	***0***	*2*	*16*	231	27	EK17	227	51
EK06	*1*	***0***	***0***	***0***	*8*	149	120	EK18	167	111
EK07	*1*	*2*	*3*	*6*	30	99	137	EK19	165	113
EK08	*5*	***0***	***0***	*4*	*14*	73	182	EK20	170	108
EK09	*2*	*2*	42	114	65	38	*15*	EK21	141	137
EK10	*1*	*9*	*7*	52	86	86	37	EK22	102	176
EK11	*19*	20	29	35	39	44	92	EK23	160	118
EK12	*7*	*5*	*15*	24	63	100	64	EK24	31	247
EK25	***0***	*10*	*11*	*9*	67	118	63	EK26	*5*	273

P. denotes points.

Categories with few participants (N < 20) are shown in italics, and categories with no participants are shown in bold. These categories were merged with adjacent categories, starting from the bottom (i.e., at zero points). The gray background indicates which categories have been merged.

For item EK25, the three-point category was merged with the two categories below it. The six-point category for item EK09 was initially left unchanged. The dichotomous items EK13 and EK26 were removed from the scale because fewer than 20 people had scored zero points.

With these modified items, the partial credit model could initially be estimated. Sum normalization was used so that the mean of item difficulties (β-parameter) was fixed at zero (Koller et al., 2012; Mair et al., 2021). This first version of the scale had a low person-separation reliability of *rel*_*P*_ = 0.469.

To test if the score categories of the ranking tasks were correctly ordered, we followed a procedure suggested by Wetzel and Carstensen (2014). For each item, we tested via *t*-tests whether the abilities (estimated from the complete scale) of those persons who had scored higher on the item were, on average, significantly higher than the abilities of those persons who had achieved one point less on the item. This was done to ensure that only items and categories with relevant information on the measured construct are included in the final test—and no items or categories where the participants' result is mostly dependent on chance. At first, we ran these *t*-tests at the α = 0.10-level to exclude the least useful items first. If the test was not significant—indicating no significant information for discriminating people's ability—the concerned adjacent categories were merged. The partial credit model was then re-estimated with the changed item categories and the *t*-tests repeated. When all *t*-tests became significant at the α = 0.10-level, they were rerun at the α = 0.05-level and the categories were adjusted until all *t*-tests became significant. We did not adjust the α-level for multiple testing during this procedure, which is the more conservative approach here: as just one failed test lead to the exclusion of the category and the item had to pass the test every time, the effective α-level decreases with the number of tests. This differs from the usual cases, where it is necessary to adjust the α-level for multiple testing.

Through this process, four items were removed (EK01, EK17, EK18, EK20) because no significantly different scoring categories remained. The person-separation reliability increased to *rel*_*P*_ = 0.564.

#### Fit analysis

We then considered the residual-based fit statistics (MSQs) for the remaining items to assess their fit to the partial credit model (Wright and Masters, 1982; Wu and Adams, 2013): the so-called Outfit (unweighted mean squares) and Infit (weighted mean squares). The expected value for both fit statistics is one. A value >1 means that there is more variance in the data than would be expected based on the model (so-called underfit; Bond and Fox, 2007). Items that show underfit discriminate worse than the other items in the test and should be removed (Wu and Adams, 2013). MSQs are dependent on sample size (Wu and Adams, 2013). The cutoff values for an acceptable fit with respect to underfit can be calculated using the formula 1 + 2 * $\sqrt{\frac{2}{N}}$ (Wu and Adams, 2013). For *N* = 278, this yields a cutoff value of 1.17. Furthermore, MSQ values can be standardized into values that follow a *t*-distribution. A *t*-value of 2 can be used as a cutoff for acceptable fit (Bond and Fox, 2007; Wright and Masters, 1982; Wu and Adams, 2013).

In this study, no item exceeded the critical threshold of MSQ > 1.17, but some items (EK23, EK09, EK06) had a *t*-value > 2. We found that successively removing these items raised the reliability. After removing the item with the worst fit, the model was re-estimated and again the item with the worst fit was removed, until reliability did not improve by removing items. Thus, items EK23, EK09, EK06, EK12, EK19, and EK24 were successively removed, ultimately increasing reliability to *rel*_*P*_ = 0.632.

For the excluded ranking tasks, we exploratively examined whether they contained single pair comparisons that exhibited good measurement properties (i.e., good differentiation between high and low ability). For this purpose, a dichotomous Rasch model was estimated for the entire scale of single pair comparisons. Seven promising pairwise comparisons were added to the partial credit model. However, most of them showed a poor fit. These were successively removed as described above to optimize reliability. Finally, only two comparisons were retained: item EK09_01-02 (comparison of oat drink vs. reusable cups) and item EK12_03-04 (comparison of lowering temperature vs. shock ventilation). Person-separation reliability increased further with these added items to *rel*_*P*_ = 0.655.

#### Tests for subgroup invariance

We conducted Andersen likelihood ratio tests (LRTs; Andersen, 1973) to further test the validity of the partial credit model with respect to differential item functioning (DIF).

We used the four split criteria of person abilities (median split), gender (female vs. non-female), age (median split), and questionnaire version (mobile version or desktop version). The global significance level was set at α = 0.10 and Bonferroni-adjusted for the individual tests, resulting in α = 0.025 (Koller et al., 2012).

The LRT was significant for age [$\chi_{\left(24\right)}^{2}$ = 46.42, *p* = 0.004] and marginally significant for gender [$\chi_{\left(24\right)}^{2}$ = 38.77, *p* = 0.029). Thus, for age and gender, Wald tests were performed, which test for model violations at the item level. As 25 item parameters were tested with respect to two split criteria, the significance level was adjusted to $\alpha \;=\;\frac{0.1}{2^{*}25}\;=\;0.002$ (Koller et al., 2012). At this level, items EK02 (threshold 1: *z* = 3.35, *p* < 0.001) and EK07 (threshold 2: *z* = −3.29; *p* = 0.001) showed significant DIF regarding age, with the former (positive z-score) being easier for younger and the latter (negative z-score) being easier for older participants. All items were retained for further analyses because the DIF was relatively well balanced (i.e., did not unilaterally disadvantage one group) and exclusion of the affected items would have lowered reliability from *rel*_*P*_ = 0.655 to *rel*_*P*_ = 0.612.

## Results

### New effectiveness knowledge scale

Table 3 shows an overview of item and person statistics for the final version of the new effectiveness knowledge scale in comparison to two intermediate states of the scale analysis and the scale by Roczen et al. (2014). We used Feldt (1980) test for comparing two Cronbach's alpha reliability coefficients to test whether the person separation reliability of the new scale (*rel*_*P*_ = 0.655) is significantly higher than the reliability of the established scale (*rel*_*P*_ = 0.329). This confirmed H1 [*t*_(276)_ = 6.14, *p* < 0.001).

**Table 3:** Characteristics of the effectiveness knowledge scale.

	**Effectiveness knowledge new scale**			**Effectiveness knowledge established scale**
	**Version without adjustments**	**Version with merged categories**	**Optimized version**	
Number of items	24	20	**16**	29
Rasch model type	PCM	PCM	**PCM**	RM
Person separation reliability	0.469	0.564	**0.655**	0.329
**Descriptive statistics**				
**Items**				
Difficulty β: *M (SD)*	0 (0.87)	0 (1.02)	**0 (1.04)**	0 (1.33)
Threshold parameter *δ: M (SD)*	0.13 (0.86)	0.12 (1.03)	**0.16 (1.07)**	–
**Persons**				
Ability *θ: M (SD)*	0.26 (0.37)	0.29 (0.59)	**0.36 (0.76)**	0.57 (0.55)
**Fit statistics**				
**Items**				
Infit *MSQ: M (SD)*	0.98 (0.07)	0.97 (0.09)	**0.97 (0.08)**	0.98 (0.06)
Infit *MSQ: min; max*	0.83; 1.10	0.82; 1.10	**0.83; 1.09**	0.90; 1.09
Infit *t:M (SD)*	−0.28 (1.21)	−0.21 (1.62)	**−0.45 (1.36)**	−0.25 (1.24)
Infit *t: min; max*	−2.46; 1.76	−3.34; 2.26	**−2.85; 1.83**	−3.23; 2.16
**Persons**				
Infit *MSQ: M (SD)*	0.94 (0.31)	0.95 (0.25)	**0.94 (0.30)**	0.98 (0.21)
Infit *t:M (SD)*	−0.17 (0.99)	−0.16 (0.90)	**−0.14 (0.91)**	−0.09 (1.01)
Persons with poor fit (*z* > 1.96)	1.80%	0.72%	**0.72%**	2.88%

All results for correlations and regression analyses reported for the new effectiveness knowledge scale are for the optimized version of the scale, denoted in bold in this table.

We asked the participants which of the two knowledge scales they liked better. 46.8% of the subjects preferred the new effectiveness knowledge scale. In contrast, 23.7% preferred the established one and 29.5 % expressed no preference. The new scale was commended for being more interactive due to the ranking tasks, and for the fact that the questions were more varied, interesting, precise, easier, and closer to everyday life. However, subjects criticized the higher completion time and text volume of the new scale.

### Results of testing the validation hypotheses

To test hypotheses 2, 4, 5, and 7, we calculated Pearson-correlations between the variables. These were tested for robustness by calculating a winsorized correlation (with 10% of the smallest and largest scores being winsorized). Inter-correlations of the most important variables are displayed in Table 4. Person abilities measured by the new effectiveness knowledge scale correlated at *r* = 0.40 (*p* < 0.001, 95% CI [0.29, 0.49]) with those measured by the established scale (H2). This correlation is limited at the upper end due to low reliabilities. Thus, correction for measurement error attenuation increased the correlation to *r* = 0.86. However, it needs to be noted that the items EK07 and EK16 appear in both scales. If they are removed from both scales, the correlation is only *r* = 0.29 (*p* < 0.001, 95% CI [0.18, 0.39]), and *r* = 0.70 if corrected for measurement error attenuation. The winsorized correlation is *r* = 0.26 (*p* < 0.001, 95% CI [0.15, 0.37]). Thus, H2 was confirmed.

**Table 4:** Correlation matrix.

	**Variable**	**1**	**2**	**3**	**4**
1	Effectiveness knowledge new scale		0.86	0.21	0.39
2	Effectiveness knowledge established scale	0.40^***^		0.36	0.36
3	Environmental attitude	0.16^**^	0.19^**^		0.66
4	High-impact PEB	0.26^***^	0.17^**^	0.51^***^	

^**^p < 0.01.

^***^p < 0.001.

Figures below the diagonal are uncorrected Pearson correlations, while those above the diagonal are corrected for measurement error attenuation, using the ratio between the observed correlation and the square root of the product of the two reliabilities (cf. Charles, 2005).

As postulated in H4, effectiveness knowledge and high-impact PEB correlated moderately at *r* = 0.26 (*p* < 0.001, 95% CI [0.15, 0.37]). The winsorized correlation was slightly lower (*r* = 0.21, *p* < 0.001, 95% CI [0.09, 0.32]).

Descriptively, the correlation between high-impact PEB and the new scale is higher than with the established scale (see Table 4: *r* = 0.26 vs. *r* = 0.17). However, this difference is not significant (*z* = 1.40, *p* = 0.081), using Hittner et al.'s (2003) variant of Dunn and Clark's z (1969). Thus, H5 is rejected.

Contrary to H7, we found that effectiveness knowledge and environmental attitude were only weakly correlated (*r* = 0.16, *p* = 0.004, 95% CI [0.04, 0.27]).

**Figure 2:**
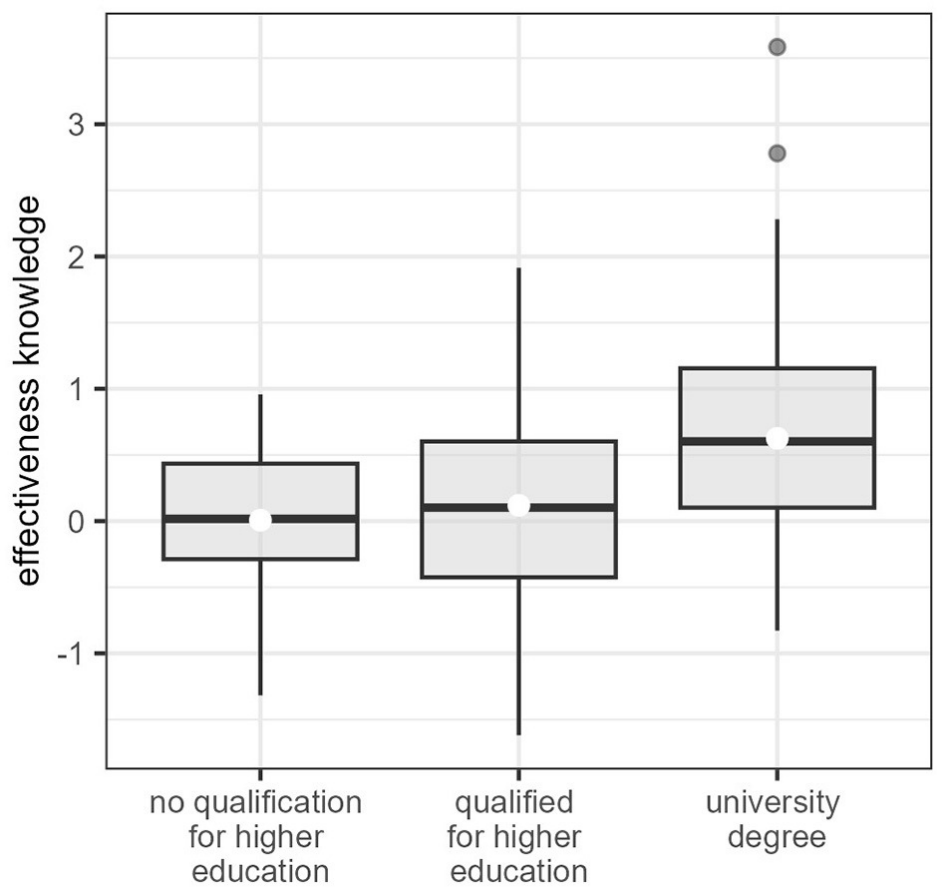
Boxplots to visualize the differences in effectiveness knowledge by educational level. The boxplots visualize the distribution of effectiveness knowledge for each of the three educational groups. The horizontal thick line marks the median, and the white dots the mean of each group. The lower and upper hinges of the box correspond to the first and third quartiles. The whiskers extend from the hinge to the largest/lowest value no further than 1.5^*^ IQR from the hinge (where IQR is the inter-quartile range). Outliers, i.e., data beyond the end of the whiskers, are plotted individually.

To test H3, we used a one-way ANOVA to test for differences in effectiveness knowledge between three educational groups (see Table 1). The low-education-group had a mean knowledge score of *M* = 0.01 (SD = 0.51, 95% CI [−0.18, 0.20]). The medium-education-group had a mean score of *M* = 0.12 (SD = 0.74, 95% CI [−0.02, 0.26]), and the high-education-group had a mean score of *M* = 0.63 (SD = 0.72, 95% CI [0.51, 0.75]). The ANOVA revealed a significant difference between at least two groups (*F*_2, 273_ = 19.61, *p* < 0.001). Thus, a Tukey test was performed. This revealed that the high-education-group had a significantly higher effectiveness knowledge score than the low-education-group (*p* < 0.001; *d* = 0.90; 95% CI [0.67, 1.13]) and the medium-education-group (*p* < 0.001; *d* = 0.70; 95% CI [0.52, 0.88]). The differences remained significant after two outliers with extremely high knowledge in the high-education-group were removed (*d* = 0.92 and *d* = 0.68, respectively). For boxplots of the effectiveness knowledge in the three groups see Figure 2.

For the regression analysis, we excluded one influential case with an increased Cook's distance in comparison to the other cases, and a leverage >0.20 (Field et al., 2012; Hemmerich, n.d.; Walther, 2020). The homoscedasticity of the residuals was slightly violated in all regressions. These violations may lead to biases in the estimated standard errors, which can lead to errors in inferential statistical testing (Eid et al., 2015, p. 720).

Contrary to H6 and as shown in Table 5, we did not find a moderating influence of environmental attitude on the relationship between effectiveness knowledge and high-impact PEB.

**Table 5:** Regression of environmental behavior on environmental attitude and effectiveness knowledge.

**Variable**	**New knowledge scale**			**Established knowledge scale**		
	β	***t***	***p***	β	***t***	***p***
**Base model without knowledge**						
EA	0.52 [0.41, 0.62]	9.74	< 0.001			
Overall model	*R*^2^ = 0.258, $R_{adj}^{2}=0.255$					
	*F*_1, 273_ = 94.77, *p* = 0.001					
	AIC = 704.04, BIC = 714.89					
**Model with knowledge and with interaction effects**						
EA	0.49 [0.38, 0.60]	9.06	< 0.001	0.52 [0.41, 0.63]	9.39	< 0.001
EK	0.18 [0.08, 0.28]	3.40	< 0.001	0.08 [−0.03, 0.18]	1.44	0.15
EA ^*^ EK	0.05 [−0.06, 0.16]	0.89	0.38	0.07 [−0.02, 0.17]	1.48	0.14
Overall model	*R*^2^ = 0.291, $R_{adj}^{2}=0.283$			*R*^2^ = 0.269, $R_{adj}^{2}=0.261$		
	*F*_3, 271_ = 37.03, *p* = 0.001			*F*_3, 271_ = 33.23, *p* = 0.001		
	AIC = 695.52, BIC = 713.61			AIC = 703.84, BIC = 721.93		

EA, Environmental attitude; EK, Effectiveness knowledge; AIC, Akaike information criterion; BIC, Bayesian information criterion.

Only standardized regression weights are given. Values in brackets are the lower and upper bounds for the 95% intervals of confidence.

As expected in H8, adding the new effectiveness knowledge scale to the regression model significantly increased the proportion of explained variance ($\Delta R_{adj}^{2}\;=\;0.028$, *F*_2, 271_ = 6.31, *p* = 0.002). In contrast, the addition of the established scale did not lead to a higher proportion of explained variance ($\Delta R_{adj}^{2}\;=\;0.006$, *F*_2, 271_ = 2.09, *p* = 0.13), confirming H9. The AIC and BIC, which put a greater emphasis on the parsimony of a model, also indicate additional explanatory power for the new scale but not for the established one: for the new scale, both criteria indicate a model which includes environmental attitude and knowledge but no interaction term as the best model (AIC = 694.32; BIC = 708.79) with a meaningful improvement over the base model (ΔAIC = 9.72; ΔBIC = 6.10). For the established scale, the best model according to BIC is the baseline model, and the best model according to AIC is only marginally better than the base model (ΔAIC = 0.20). This lends further support to H8 and H9, and the rejection of H6.

## Discussion

### Scale construction

The goal of this study was to develop a new scale to measure effectiveness knowledge with a separation reliability of at least 0.70 fitting a partial credit Rasch model. While the data fits a partial credit model quite well (see Table 3), only a reliability of *rel*_*P*_ = 0.655 was reached. Nevertheless, the reliability of the newly developed scale is still higher than what has been achieved in previous studies on the construction of an effectiveness knowledge scale (Díaz-Siefer et al., 2015; Frick, 2003; Frick et al., 2004) and significantly higher than the reliability of Roczen et al. (2014) scale in this sample (*rel*_*P*_ = 0.329; H1 confirmed). With 16 items, the scale is shorter than the scales found in the literature, which makes it easier to use in practice. The surveyed test subjects also predominantly preferred the new scale over the established one.

It is not possible to say conclusively whether the goal of developing an easier scale than the previous ones was achieved, as the sample had an above-average level of education (see Table 1), and even the established scale from Roczen et al. (2014) tended to be too easy (see Table 3). The new scale was slightly more difficult, but only after summarizing thresholds in the lower ability range. In general, the new scale seems suitable for a broader ability spectrum, as the PCM allows adaptation of the difficulty by summarizing categories in which few persons are located.

Guessing is probably a factor in the lower than expected reliability of the scale: the instruction explicitly allowed guessing, as this was intended to prevent results from being influenced by the individual tendency to guess. However, this guessing leads to the fact that persons who actually knew the correct answer can no longer be distinguished from persons who found the correct answer by guessing. Accordingly, guessing lowers the reliability of a test (Paek, 2015).

On the other hand, the low reliability, which was also found by other researchers developing scales on effectiveness knowledge (see above), could also be an indication of an underlying multidimensionality of the construct. The dimensions could, for example, be differentiated along the content-related sub-areas (knowledge about mobility, knowledge about nutrition, about energy, etc.). What they have in common is perhaps not the construct of effectiveness knowledge at all, but only crystallized intelligence (see Geiger et al., 2019). In future studies, the question of dimensionality should be investigated by constructing further items for assumed content-related sub-domains and investigating the dimensional structure with factor-analytical or multidimensional IRT approaches.

### Scale validation

As expected in H2, we found a moderate (or high if corrected for measurement error attenuation) correlation between the test scores on the newly developed and the established effectiveness knowledge scale. This is an indication of convergent validity of the new scale.

The relationship between effectiveness knowledge and educational level (H3) was shown in the respect that individuals with a university degree showed significantly higher knowledge scores than individuals without a university degree. The effects found are moderate to large, and within the range of what previous studies have found (Cologna et al., 2022; Díaz-Siefer et al., 2015). This is a further indication of construct validity.

As expected in H4, we found a significant, weak to moderate relationship between effectiveness knowledge and high-impact environmental behavior. However, contrary to our expectations, this correlation was not significantly higher for the new scale compared to the established scale (H5). This is likely due to the fact that the power of the present study was not high enough to detect differences between low to medium correlations.

Contrary to H6, we did not find a moderating influence of environmental attitude on the knowledge-behavior relationship. Instead, we found two distinct main effects of environmental attitude and effectiveness knowledge on high-impact environmental behavior, with the former effect being larger. Assuming a causal relationship, this would mean that there are two ways to promote high-impact PEB: via fostering effectiveness knowledge and via environmental attitude. Individuals with high environmental attitude might make climate-friendly decisions even without high effectiveness knowledge. Perhaps they do not distinguish between high- and low-impact behavior but behave in a more climate-friendly way in all domains (Bruderer Enzler and Diekmann, 2019; Gatersleben et al., 2002). It is more difficult to explain why individuals with high knowledge but low environmental attitude should nevertheless behave in a climate-friendly manner. This finding, however, is probably an artifact of a very environmentally conscious sample: On a scale of one to five, only 51 individuals (18.48%) have an environmental attitude score lower than four, and only four individuals have a score lower than three (*M* = 4.35). Thus, the environmental attitude in our sample is significantly higher than in the overall German population (Belz et al., 2022; Rubik et al., 2019; Stieß et al., 2022). It is highly probable that the expected interaction could not be found because the variance on the construct environmental attitude was too low, i.e. people in the lower attitude spectrum were missing in our sample. We therefore suggest interpreting our findings not as general evidence on the moderation of the effect of effectiveness knowledge on high-impact PEB through environmental attitude, but rather as evidence that effectiveness knowledge has a significant effect on high impact PEB in samples with high environmental attitude.

Contrary to H7, we found only a weak relationship between environmental attitude and effectiveness knowledge. This could also partly be due to the ceiling effect in environmental attitude (Glen, 2015). However, since environmental attitude correlates highly with environmental behavior (which shows similar reliability and similar distribution as effectiveness knowledge), this cannot be the only reason. Nevertheless, the correlation magnitude in this study is similar to the correlation of *r* = 0.18 found by Meinhold and Malkus (2005) between environmental attitude and environmental knowledge (confounded with self-assessment).

Effectiveness knowledge, measured by the new scale, explained nearly 3% more variance in high-impact environmental behavior than environmental attitude alone, indicating incremental validity (H8). In contrast, the established scale by Roczen et al. (2014) showed no significant contribution to explaining environmental behavior beyond environmental attitude (H9). Although 3% of additional variance might not seem like much, we must stress that this is additional explained variance for behavior with high impact on climate change, so 3% on behaviors that have very direct consequences on one of the most urgent crises humanity is facing right now.

Also, the homogeneity of the sample and possible self-report bias may lead to an underestimation of the effect in the general population: environmental attitude and high-impact PEB were measured by self-report, which makes these scales susceptible to bias (Kormos and Gifford, 2014; Nielsen et al., 2022). This could lead to an overestimation of the relationship between environmental attitude and high-impact PEB (through the common factor of social desirability), and to an underestimation of the relationship of these constructs with effectiveness knowledge, as knowledge tests are less susceptible to self-report-bias. Cheating poses a risk for knowledge tests, but the instruction asked not to research answers and nothing was at stake for the participants, so we assume that cheating rates are low in our sample.

### Limitations

The sample studied was not representative of the population in Germany—indicated, for example, by the high level of education and environmental attitude. Descriptive findings, such as the effectiveness knowledge level in this study being rather high, can therefore not be generalized (Leiner, 2016). The relative ordering of items with respect to their difficulty, however, should be equally evident in other samples, due to the properties of the Rasch model. The extent to which the correlations found can be generalized cannot be answered unequivocally: studies indicate that findings on relationships between variables are less susceptible to bias due to non-representativeness of the sample than descriptive findings on distributions, such as means (Diekmann, 2007; Leiner, 2016). However, this only holds true if there is no restriction of variance, which can be doubted for the present study at least for environmental attitude (see above).

Hence, both the difficulty of the new effectiveness knowledge scale and the validation hypotheses should be tested on a more representative sample in future studies. Possibly, some of the items that were too easy in our study and were consequently removed would be more suitable in other samples. As some subjects criticized the higher completion time and text volume of the new scale, it should also be critically examined whether some item formulations could be linguistically simplified.

The knowledge items were created based on current greenhouse gas-related life cycle analyses, predominantly from Germany. Thus, they are not fully transferable to other local and temporal contexts. For many items this is not much of a problem, e.g., tofu will likely always be more climate-friendly than beef, irrespective of locality. However, for items comparing actions from different sectors (e.g., driving compared to meat consumption), shifts could occur if the energy mix becomes increasingly climate-friendly, or if a country has a significantly different energy mix than Germany. This should therefore be critically examined before using the scale.

Another practical limitation of the new scale results from the individual threshold summary. This eliminated the problem of interchanged categories (Wetzel and Carstensen, 2014) and increased the reliability of the scale. However, if the scale is used in a new sample, the swapped or not significantly distinguishable categories may not be the same. The need for category merging in our sample was probably driven by the relatively small sample size and high level of education, resulting in some empty or sparse categories. A simple scoring scheme can only be created for the first step of the evaluation (see Figure 1). Subsequently, the category order should be checked for each sample and adjustments may have to be made. This might pose a practical hurdle for the application of the scale in future research practice.

A more practical approach might be creating a scale based on multiple pairwise comparisons of two options each instead of ranking tasks. This remains a promising approach for future research. However, it should be considered that this results in a high number of items, and that the Rasch model's assumption of local stochastic independence of the items is violated if the same object is used in multiple comparisons.

The scale's scope is limited to impacts on climate change and does not consider other areas of environmental behavior addressing e.g. pollution or biodiversity loss. In this aspect, our scale differs from the established scale used for testing convergent validity (Roczen et al., 2014). Therefore, it may be more accurate to describe the knowledge measured with the new scale as *climate impact knowledge* rather than *environmental effectiveness knowledge*. However, as these concepts are strongly related and the latter term is already established in the literature, we don't deem it useful to introduce yet another term into the field. Furthermore, the scale primarily captures knowledge about the climatic effects of individual (consumption) behavior because there is a good data base in this area to construct items on. The primary dependent variable in this study also primarily reflects individual behavior. However, while individual behavioral changes can be a building block in addressing the climate crisis, they are not sufficient on their own (Matthies, 2018; Verfuerth et al., 2019). Further studies could attempt to expand the content of the scale to include climate protection policies or develop similar scales for other areas of environmental impact, such as the protection of biodiversity.

Considering the high-impact-PEB scale used for validation, it might be more precise to use actual carbon footprints (Gatersleben, 2012; Nielsen et al., 2022). However, measuring these accurately comes with a lot of effort for both researchers and participants. We consider our approach a good compromise, but encourage future researchers to measure carbon footprints more precisely if resources are available.

## Conclusion

Various studies have shown people's knowledge deficits in assessing the climate impacts of various actions (Attari et al., 2010; Cologna et al., 2022; Gardner and Stern, 2008; Tofighi and Jackson, 2020; Wynes et al., 2020). In our study, we were able to develop a scale that captures this form of knowledge by using ranking tasks and the partial credit model. Despite some weaknesses (see above), it can measure this knowledge much more accurately than the established scale by Roczen et al. (2014). It is also able to predict some additional variance in high-impact pro-environmental behavior, a domain where every single percent is important, as even small reductions in high-impact behavior in only a subset of the population can have a meaningful effect on the climate.

This study demonstrates that it is possible to improve the measurement of effectiveness knowledge both in reliability and predictive validity by using ranking tasks on the order of climate impact of various options instead of asking for concrete numbers via multiple choice items.

While the contents of some items will need adaptions for use outside of Germany due to differences in the energy mix, the type of item itself should work in other countries as well—although this remains to be tested empirically by further research. The list of items tested and found useful for measuring effectiveness knowledge in Germany is found in Supplementary Appendix A1 in English and German. Feel free to use or adapt them for your research.

## Data Availability

The raw data supporting the conclusions of this article will be made available by the authors, without undue reservation. If you are interested, please send the corresponding author an email.
