# Supplementary material for: Better climate action through the right knowledge? Development and validation of an item-response-theory scale measuring climate effectiveness knowledge
Source: Front Psychol. 2024 Nov 14;15:1347407. doi: 10.3389/fpsyg.2024.1347407 (PMC11605514; doi:10.3389/fpsyg.2024.1347407)
Supplement: Supplementary file 1 [file Table_1.pdf]

## Appendix A1: New Effectiveness Knowledge Scale

Please note that the scale was developed and validated only in the German version (see Table A1.2)

**Table A1.1**

*Items of the new effectiveness knowledge scale, English translation*

*Ranking tasks*

**EK01 *(deleted)***

How climate-friendly or -harmful are the following foods? Please sort them according to the amount of greenhouse gas emissions that occur during their life cycle (i.e., for production, packaging, and transport; for 1kg of food each) until they are in our supermarket.

*(1 = most climate-friendly; 4 = most climate-harmful)*

1. potatoes → *0,2kg $CO_2$-eq / kg (Reinhardt et al., 2020)*
2. pasta → *0,7kg $CO_2$-eq / kg (Reinhardt et al., 2020)*
3. lentils → *1,2kg $CO_2$-eq / kg (Reinhardt et al., 2020)*
4. rice → *3,1kg $CO_2$-eq / kg (Reinhardt et al., 2020)*

**EK02**

You are preparing a lasagna: How many greenhouse gas emissions can be saved by the following measures? Please sort.

*(1 = largest savings; 4 = smallest savings)*

1. substituting beef with soy pellets → *– 900 g $CO_2$-eq / portion (Reinhardt et al., 2020)*
2. substituting beef with pork → *– 600 g $CO_2$-eq / portion (Reinhardt et al., 2020)*
3. substituting cheese with vegan gourmet slices (coconut fat based)
   → *– 100 g $CO_2$-eq / portion (Reinhardt et al., 2020)*
4. substituting canned strained tomatoes with strained tomatoes from a glass bottle
   → *constant or even slightly worse (Reinhardt et al., 2020)*

**EK03**

How climate-friendly or -harmful are the following foods? Please sort them according to the amount of greenhouse gas emissions that occur during their life cycle (i.e., for production, packaging, and transport; for 1kg of food each) until they are in our supermarket.

*(1 = most climate-friendly; 4 = most climate-harmful).*

1. carrots → *0,1 kg $CO_2$-eq / kg (Reinhardt et al., 2020)*
2. tofu → *1,0 kg $CO_2$-eq / kg (Reinhardt et al., 2020)*
3. chicken → *5,5 kg $CO_2$-eq / kg (Reinhardt et al., 2020)*
4. beef → *13,6 kg $CO_2$-eq / kg (Reinhardt et al., 2020)*

**EK04**

Which of the following actions can save the most greenhouse gas emissions (on average) over a year? Please rank the actions according to their savings potential.

*(1 = largest savings; 4 = smallest savings)*

1. Abolishing your own car → *1,4 – 1,5 t $CO_2$-eq / year (KlimAktiv, 2020)*
2. Switching to a vegetarian diet (this means to stop eating meat, but not dairy products and eggs)
   → *0,44 t $CO_2$-eq / year (KlimAktiv, 2020)*
3. Taking warm showers only every other day instead of every day
   → *0,25 t $CO_2$-eq / year (Verbraucherzentrale Nordrhein-Westfalen e.V., 2017)*
4. Completely avoiding plastic packaging
   → *0,12 t $CO_2$-eq / year (Initiative Psychologie im Umweltschutz e.V [IPU], o. D.), o. D.)*

*(Table is continued on next page.)*

**Table 1.1 (continued)**

**EK05**

You want to travel from Cologne to Berlin. How climate-friendly or climate-harmful are the possible means of transport? Please rank the four means of transport according to their greenhouse gas emissions.

Note: We are looking for emissions per passenger kilometer at an average occupancy rate. In addition to the emissions caused using the vehicle, the emissions for the provision of energy (e.g., electricity, gasoline), for the production of the vehicle and its disposal at the end of its life cycle must also be included proportionally.

*(1 = most-climate-friendly; 4 = most climate-harmful)*

1. long-distance bus → *31,48 g $CO_2$-eq / passenger kilometers (pkm) (Allekotte et al., 2021)*
2. long-distance train → *33,93 g $CO_2$-eq / pkm (Allekotte et al., 2021)*
3. car → *189,4 g $CO_2$-eq / pkm (Allekotte et al., 2021)*
4. domestic flight → *215,69 g $CO_2$-eq / pkm (Allekotte et al., 2021)*

**EK06 *(deleted)***

There are many ways to get around in the city. How climate-friendly or climate-harmful are they? Please rank the four modes of transportation according to their greenhouse gas emissions.

Note: We are looking for emissions per passenger kilometer at an average occupancy rate. In addition to the emissions caused using the vehicle, the emissions for the provision of energy (e.g., electricity, gasoline), for the production of the vehicle and its disposal at the end of its life cycle must also be included proportionally.

*(1 = most climate-friendly; 4 = most climate-harmful)*

1. normal bike → *8,68 g $CO_2$-eq / pkm (Allekotte et al., 2021)*
2. e-bike/pedelec → *14,52 g $CO_2$-eq / pkm (Allekotte et al., 2021)*
3. tram, city railway or subway → *64,11 g $CO_2$-eq / pkm (Allekotte et al., 2021)*
4. car → *189,4 g $CO_2$-eq / pkm (Allekotte et al., 2021)*

**EK07**

Please sort the following light bulbs by their energy efficiency (i.e., how much electricity they use to produce the same amount of light).

*(1 = most efficient; 4 = most inefficient)*

1. LED (light emitting diodes) → *120 lm / W (Deutsche Energie-Agentur GmBH [dena], 2018)*
2. energy-saving bulb (compact fluorescent lamp) → *60 lm / W (dena, 2018)*
3. halogen lamp → *15 lm / W (dena, 2018)*
4. conventional light bulb → *12 lm / W (dena, 2018)*

**EK08**

How climate-friendly or climate-harmful are the following energy sources?

Please sort the energy sources according to their greenhouse gas emissions (CO2 equivalent per Kilowatt hour).

*(1 = most climate-friendly; 4 = most climate-harmful)*

1. wind → close to *0 g $CO_2$-eq / KWh (UBA, 2021)*
2. natural gas → *201 g $CO_2$-eq / KWh (Bundesamt für Wirtschaft und Ausfuhrkontrolle [BAFA], 2020)*
3. diesel → *266 g $CO_2$-eq / KWh (BAFA, 2020)*
4. lignite → *383g $CO_2$-eq / KWh (BAFA, 2020)*

*(Table is continued on next page.)*

**Table 1.1 (continued)**

**EK09**

You go to the bakery and get a latte and a sandwich. Which decision will save the most greenhouse gas emissions? Please sort the four possible actions by their potential savings.

*(1 = largest savings; 4 = smallest savings)*

1. coffee with oat drink instead of milk (assumption: 100ml milk or oat drink)
   → *– 100 g $CO_2$-eq (Reinhardt et al., 2020)*
2. coffee in reusable instead of disposable cups
   → *– 40 g $CO_2$-eq (maximum; Deutsche Umwelthilfe e.V [DUH], 2015)*
3. no sugar in coffee (assumption: 0g sugar instead of 10g sugar) **– *(deleted)***
   → *– 7 g $CO_2$-eq (Reinhardt et al., 2020)*
4. sandwich with cheese instead of sausage (from pork) **– *(deleted)***
   → *+ 56 g $CO_2$-eq (assumption: 1 slice of 20g; Reinhardt et al., 2020)*

**EK10**

Please sort the following actions by how many greenhouse gas emissions they emit.

*(1 = most climate-friendly; 4 = most climate-harmful)*

1. illuminate an apartment for three hours a day for one year with 30 LED lamps
   → *100 kg $CO_2$-eq (with 7-watt lamps, German electricity mix; Icha, 2021)*
2. eat 150g of meat every day for one year (beef, pork, and chicken in equal parts)
   → *430 kg $CO_2$-eq (Reinhardt et al., 2020)*
3. one vacation trip with an intra-European flight, totaling 3,500 km (roughly equivalent to a round trip to Portugal)
   → *700 kg $CO_2$-eq (Allekotte et al., 2021)*
4. commuting to work every workday for one year with an average combustion car at a distance to the place of work of 10.5km (corresponds to the median in Germany)
   → *1020 kg $CO_2$-eq (Allekotte et al., 2021)*

**EK11**

Please sort the following countries by their per capita greenhouse gas emissions (per year).

*(1 = lowest; 4 = highest)*

1. India → *2,31 t $CO_2$-eq / Person (Climate Watch, 2021)*
2. China → *9,62 t $CO_2$-eq / Person (Climate Watch, 2021)*
3. Germany → *10,37 t $CO_2$-eq / Person (Climate Watch, 2021)*
4. USA → *20,69 t $CO_2$-eq / Person (Climate Watch, 2021)*

**EK12 *(deleted)***

You live in an old single-family house (140 square meters, about 50 years old) that has not been renovated. How much do the following measures help to reduce heating energy consumption? Please sort.

*(1 = largest savings; 4 = smallest savings)*

1. insulate the roof ***(deleted)*** → *– 450 kg $CO_2$-eq / year (KlimAktiv, 2020)*
2. equip all windows with heat-insulating glass ***(deleted)***
   → *– 360 kg $CO_2$-eq / year (KlimAktiv, 2020b)*
3. reduce the average room temperature by 1°C
   → *– 300 kg $CO_2$-eq / year (KlimAktiv, 2020)*
4. do not leave the windows tilted for a long time, but open them fully for some minutes instead.
   → *– 270 kg $CO_2$-eq / year (KlimAktiv, 2020)*

*(Table is continued on next page.)*

**Table 1.1 (continued)**

**EK25**

Please sort the following things according to how much they benefit or harm the climate (each calculated over one year).

*(1 = greatest benefit to the climate; 4 = greatest harm to the climate)*

1. a speed limit on German highways of 130 km/h (instead of no speed limit)
   → *approx. - 1.9 million t $CO_2$-eq / year (Lange, 2020)*
2. ten soccer fields of forest (instead of inanimate wasteland)
   → *approx. - 80 t $CO_2$-eq / year (Bundesinformationszentrum Landwirtschaft [BLE], o. D.)*
3. all food wasted in Germany (the greenhouse gas emissions generated on the life cycle, i.e., for production, packaging, and transport of all those foods that are wasted)
   → *approx. + 48 million t $CO_2$-eq / year (Noleppa & Cartsburg, 2015)*
4. all lignite-fired power plants in Germany combined
   → *approx. + 150 million t $CO_2$-eq / year (Fraunhofer ISE, 2020)*

*Single-Choice tasks*

**EK13 *(deleted)***

Which of the following actions will save the most greenhouse gas emissions?

- When heating, leave windows permanently tilted instead of opening them fully for a short time.
- **Insulate buildings well, especially roofs and windows.**
- Keep freezers free of ice.
- Provide the washing machine with its own hot water connection.

*Item adopted from Gossen et al. (2021)*

**EK14**

If the CO2 emissions, which may maximally be emitted in 2050, were equally distributed among all people currently living on earth, then a person has a CO2 budget of about 2 tons per year. How much of this budget does a person consume for a flight from Frankfurt am Main to New York (round trip)?

- about 25 % (one quarter)
- about 75 % (three quarters)
- about 100 % (the whole)
- **about 150 % (one and a half times)**

*In Economy Class, approx. 3 t $CO_2$-eq are emitted per person (atmosfair gGmbH, 2019)*

**EK15**

How many greenhouse gas emissions does one piece of butter cause compared to the same amount of margarine?

- about half as many
- about twice as many
- **about four times as many**
- about fifteen times as many

*Emissions 250 g butter: 2.25 kg $CO_2$-eq; 250 g margarine: 0.56 kg $CO_2$-eq (Reinhardt et al., 2020)*

**EK16**

An average household in Germany consumes the most energy for...

- lighting → *Electricity total: 14 % (Heizspiegel.de, 2021)*
- hot water → *15 % (Heizspiegel.de, 2021)*
- **heating** → *71 % (Heizspiegel.de, 2021)*
- household appliances and electronical devices → *Electricity total: 14 % (Heizspiegel.de, 2021)*

*Item adopted from Geiger et al. (2019)*

*(Table is continued on next page.)*

**Table 1.1 (continued)**

**EK17** ***(deleted)***

How long does a wind turbine have to run to recover the energy used to build, operate, and dismantle it?

- 2 to 6 weeks
- **3 to 7 months** *(UBA, 2021)*
- 1 to 2 years
- 4 to 5 years

**EK18** ***(deleted)***

A single flight from Frankfurt am Main to New York produces about as many greenhouse gas emissions as if an LED light (at 7 watts) ...

- shines continuously for half a year.
- shines continuously for five years.
- **shines continuously for 60 years.**
- shines continuously for 500 years.

*Flight emissions: 1.5 t $CO_2$-eq in economy class (atmosfair gGmbH, 2019); light emissions: 436 g $CO_2$-eq / KWh electricity (KlimAktiv, 2020)*

**EK19** ***(deleted)***

The greenhouse gas emissions saved by a person a day by completely eliminating animal products from their diet are equivalent to driving a car...?

- 700 meters
- 2,3 kilometers
- **9,6 kilometers** → *1.8 kg $CO_2$-eq*
- 18,5 kilometers

*Emissions from car travel: Allekotte et al. (2021); emissions from food: KlimAktiv (2020)*

**EK20** ***(deleted)***

How long would you have to live vegan to "offset" the emissions of a flight from Hamburg to Mallorca and back?

- about one month
- about five months
- **about one year**
- about two years

*Flight emissions: 617 kg - 770 kg $CO_2$-eq (atmosfair gGmbH, 2019); Savings through a vegan diet: approx. 680kg $CO_2$-eq per year (KlimAktiv, 2020)*

**EK21**

When buying fresh tomatoes in winter: Which ones should you take to emit as few greenhouse gases as possible?

- Tomatoes from Germany (heated greenhouse)
  → *2,9 kg $CO_2$-eq / kg tomato (Reinhardt et al., 2020)*
- **Tomatoes from Southern Europe / North Africa (open field)**
  → *0,4 kg $CO_2$-eq / kg tomato (Reinhardt et al., 2020)*

**EK22**

Organic meat produces fewer greenhouse gas emissions than conventional meat.

- True
- **False** *(Reinhardt et al., 2020)*

**EK23** ***(deleted)***

Which T-shirt is the most climate-friendly?

- **One made of cotton fabric** → *4,3 kg $CO_2$-eq (Kirchain et al., 2015)*
- One made of polyester fabric → *7,1 kg $CO_2$-eq (Kirchain et al., 2015)*
- One made of wool → *11 kg $CO_2$-eq (Kirchain et al., 2015)*

*(Table is continued on next page.)*

**Table 1.1 (continued)**

**EK24 *(deleted)***

Recycled paper is more climate-friendly than paper made from virgin fiber.

- **True** *(Grießhammer et al., 2010)*
- False

**EK26 *(deleted)***

Which water should you drink if you want to protect the climate?

- Water from 5-liter plastic canisters
- Water from plastic bottles
- Water from glass bottles
- **Tap water** *(a tip: tap e.V., 2021)*

*Note*. For the ranking tasks, the order given here corresponds to the correct order. In the single-choice tasks, the correct solution is indicated by bold type. Why items were deleted is explained in the methods section. The reason for this can be, for example, a poor fit, or low item information.

**Table A1.2**

*Items of the new effectiveness knowledge scale, original German version.*

*Ordnungsaufgaben*

**EK01 *(gestrichen)***

Wie klimafreundlich bzw. -schädlich sind die folgenden Lebensmittel? Bitte sortieren Sie sie nach der Menge der Treibhausgasemissionen, die auf ihrem Lebensweg (d. h. für Produktion, Verpackung und Transport; auf je 1kg Lebensmittel betrachtet) anfallen, bis sie bei uns im Supermarkt stehen.

*(1 = am klimafreundlichsten; 4 = am klimaschädlichsten)*

1. Kartoffeln → *0,2kg $CO_2$-eq / kg (Reinhardt et al., 2020)*
2. Nudeln → *0,7kg $CO_2$-eq / kg (Reinhardt et al., 2020)*
3. Linsen → *1,2kg $CO_2$-eq / kg (Reinhardt et al., 2020)*
4. Reis → *3,1kg $CO_2$-eq / kg (Reinhardt et al., 2020)*

**EK02**

Sie bereiten eine Lasagne zu: Wie viele Treibhausgasemissionen lassen sich durch die folgenden Maßnahmen sparen? Bitte sortieren Sie.

*(1 = größte Einsparung; 4 = kleinste Einsparung)*

1. Rindfleisch durch Sojagranulat ersetzen → *– 900 g $CO_2$-eq / Portion (Reinhardt et al., 2020)*
2. Rindfleisch durch Schweinefleisch ersetzen → *– 600 g $CO_2$-eq / Portion (Reinhardt et al., 2020)*
3. Käse durch vegane Genießerscheiben (auf Kokosfettbasis) ersetzen → *– 100 g $CO_2$-eq / Portion (Reinhardt et al., 2020)*
4. Passierte Tomaten aus der Dose durch passierte Tomaten aus der Glasflasche ersetzen → *gleichbleibend oder sogar etwas schlechter (Reinhardt et al., 2020)*

**EK03**

Wie klimafreundlich bzw. -schädlich sind die folgenden Lebensmittel? Bitte sortieren Sie sie nach der Menge der Treibhausgasemissionen, die auf ihrem Lebensweg (d. h. für Produktion, Verpackung und Transport; auf je 1kg Lebensmittel betrachtet) anfallen, bis sie bei uns im Supermarkt stehen.

*(1 = am klimafreundlichsten; 4 = am klimaschädlichsten)*

1. Karotten → *0,1 kg $CO_2$-eq / kg (Reinhardt et al., 2020)*
2. Tofu → *1,0 kg $CO_2$-eq / kg (Reinhardt et al., 2020)*
3. Hähnchenfleisch→ *5,5 kg $CO_2$-eq / kg (Reinhardt et al., 2020)*
4. Rindfleisch → *13,6 kg $CO_2$-eq / kg (Reinhardt et al., 2020)*

**EK04**

Durch welche der folgenden Handlungen lassen sich (im Durchschnitt) auf ein Jahr betrachtet am meisten Treibhausgasemissionen einsparen? Bitte ordnen Sie die Handlungen nach Ihrem Einsparpotential.

*(1 = größte Einsparung; 4 = kleinste Einsparung)*

1. eigenes Auto abschaffen → *1,4 – 1,5 t $CO_2$-eq / Jahr (KlimAktiv, 2020)*
2. sich vegetarisch ernähren (Damit ist ein Verzicht auf Fleisch, aber kein Verzicht auf Milch- und Eiprodukte gemeint) → *0,44 t $CO_2$-eq / Jahr (KlimAktiv, 2020)*
3. nur jeden zweiten Tag statt jeden Tag warm duschen → *0,25 t $CO_2$-eq / Jahr (Verbraucherzentrale Nordrhein-Westfalen e.V., 2017)*
4. komplett auf Plastikverpackungen verzichten → *0,12 t $CO_2$-eq / Jahr (Initiative Psychologie im Umweltschutz e.V [IPU], o. D.)*

*(Table is continued on next page.)*

**Table1.2 (continiued)**

**EK05**

Sie möchten von Köln nach Berlin reisen. Wie klimafreundlich oder -schädlich sind die möglichen Verkehrsmittel? Bitte ordnen Sie die vier Verkehrsmittel nach ihrem Ausstoß von Treibhausgasen.

Anmerkung: Gesucht sind die Emissionen pro Personenkilometer bei einer durchschnittlichen Auslastung. Einzubeziehen sind, neben den Emissionen, die bei der Nutzung des Fahrzeugs entstehen, anteilig auch die Emissionen für die Energiebereitstellung (z. B. Strom, Benzin), für die Herstellung des Fahrzeuges und dessen Entsorgung am Ende seiner Lebensdauer.

*(1 = am klimafreundlichsten; 4 = am klimaschädlichsten)*

1. Fernlinienbus → *31,48 g $CO_2$-eq / Personenkilometer (Pkm) (Allekotte et al., 2021)*
2. Bahn-Fernverkehr → *33,93 g $CO_2$-eq / Pkm (Allekotte et al., 2021)*
3. PKW → *189,4 g $CO_2$-eq / Pkm (Allekotte et al., 2021)*
4. Inlandsflug → *215,69 g $CO_2$-eq / Pkm (Allekotte et al., 2021)*

**EK06 *(gestrichen)***

Um sich in der Stadt fortzubewegen, gibt es viele verschiedene Möglichkeiten. Wie klima-freundlich oder -schädlich sind sie? Bitte ordnen Sie die vier Verkehrsmittel nach ihrem Ausstoß von Treibhausgasen.

Anmerkung: Gesucht sind die Emissionen pro Personenkilometer bei einer durchschnittlichen Auslastung. Einzubeziehen sind, neben den Emissionen, die bei der Nutzung des Fahrzeugs entstehen, anteilig auch die Emissionen für die Energie-bereitstellung (z. B. Strom, Benzin), für die Herstellung des Fahrzeuges und dessen Entsorgung am Ende seiner Lebensdauer.

*(1 = am klimafreundlichsten; 4 = am klimaschädlichsten)*

1. normales Fahrrad → *8,68 g $CO_2$-eq / Pkm (Allekotte et al., 2021)*
2. E-Bike/Pedelec → *14,52 g $CO_2$-eq / Pkm (Allekotte et al., 2021)*
3. Straßenbahn, S- oder U-Bahn → *64,11 g $CO_2$-eq / Pkm (Allekotte et al., 2021)*
4. PKW → *189,4 g $CO_2$-eq / Pkm (Allekotte et al., 2021)*

**EK07**

Bitte sortieren Sie die folgenden Leuchtmittel nach Ihrer Energieeffizienz (d. h. danach, wie viel Strom sie verbrauchen, um gleich viel Licht zu erzeugen.)

*(1 = am effizientesten; 4 = am ineffizientesten)*

1. LED (Licht emittierende Dioden) → *120 lm / W (Deutsche Energie-Agentur GmBH [dena], 2018)*
2. Energiesparlampe (Kompaktleuchtstofflampe) → *60 lm / W (dena, 2018)*
3. Halogenlampe → *15 lm / W (dena, 2018)*
4. Herkömmliche Glühlampe → *12 lm / W (dena, 2018)*

**EK08**

Wie klimafreundlich bzw. -schädlich sind die folgenden Energieträger?

Bitte sortieren Sie die Energieträger nach Ihrem Treibhausgas-Ausstoß (CO2-Äquivalent pro Kilowattstunde).

*(1 = am klimafreundlichsten; 4 = am klimaschädlichsten)*

1. Wind → *nahe 0 g $CO_2$-eq / KWh (UBA, 2021)*
2. Erdgas → *201 g $CO_2$-eq / KWh (Bundesamt für Wirtschaft und Ausfuhrkontrolle [BAFA], 2020)*
3. Diesel → *266 g $CO_2$-eq / KWh (BAFA, 2020)*
4. Braunkohle → *383g $CO_2$-eq / KWh (BAFA, 2020)*

*(Table is continued on next page.)*

**Table1.2 (continiued)**

**EK09**

Sie gehen zum Bäcker und holen sich einen Milchkaffee und ein belegtes Brötchen. Durch welche Entscheidung lassen sich am meisten Treibhausgasemissionen einsparen? Bitte sortieren Sie die vier Handlungsmöglichkeiten nach ihrem Einsparpotential.

*(1 = größte Einsparung; 4 = kleinste Einsparung)*

1. Kaffee mit Haferdrink statt Milch (Annahme: 100ml Milch bzw. Haferdrink)
   → *– 100 g $CO_2$-eq (Reinhardt et al., 2020)*
2. Kaffee im Mehrweg- statt Einwegbecher
   → *– 40 g $CO_2$-eq (höchstens; Deutsche Umwelthilfe e.V [DUH], 2015)*
3. beim Kaffee auf Zucker verzichten (Annahme: 0g Zucker statt 10g Zucker) – ***(gestrichen)***
   → *– 7 g $CO_2$-eq (Reinhardt et al., 2020)*
4. Brötchen mit Käse statt mit Wurst (vom Schwein) – ***(gestrichen)***
   → *+ 56 g $CO_2$-eq (Annahme: 1 Scheibe à 20g; Reinhardt et al., 2020)*

**EK10**

Bitte sortieren Sie die folgenden Handlungen danach, wie viele Treibhausgasemissionen sie ausstoßen.

*(1 = am klimafreundlichsten; 4 = am klimaschädlichsten)*

1. Eine Wohnung ein Jahr lang drei Stunden pro Tag mit 30 LED-Lampen beleuchten
   → *100 kg $CO_2$-eq (bei 7-Watt-Lampen, deutscher Strommix; Icha, 2021)*
2. Ein Jahr jeden Tag 150g Fleisch essen (Rind, Schwein und Huhn zu gleichen Teilen)
   → *430 kg $CO_2$-eq (Reinhardt et al., 2020)*
3. Eine Urlaubsreise mit innereuropäischem Flug, insgesamt 3.500 km (entspricht ungefähr einer Hin- und Rückreise nach Portugal)
   → *700 kg $CO_2$-eq (Allekotte et al., 2021)*
4. Ein Jahr jeden Werktag zur Arbeit mit einem durchschnittlichen Verbrenner-Auto pendeln bei einer Distanz zum Arbeitsort von 10,5km (entspricht dem Median in Deutschland)
   → *1020 kg $CO_2$-eq (Allekotte et al., 2021)*

**EK11**

Bitte sortieren Sie die folgenden Länder nach ihrem Pro-Kopf-Ausstoß an Treibhausgasen (pro Jahr).

*(1 = am niedrigsten; 4 = am höchsten)*

1. Indien → *2,31 t $CO_2$-eq / Person (Climate Watch, 2021)*
2. China → *9,62 t $CO_2$-eq / Person (Climate Watch, 2021)*
3. Deutschland → *10,37 t $CO_2$-eq / Person (Climate Watch, 2021)*
4. USA → *20,69 t $CO_2$-eq / Person (Climate Watch, 2021)*

**EK12 *(gestrichen)***

Sie leben in einem alten Einfamilienhaus (140 Quadratmeter, ca. 50 Jahre alt), welches nicht saniert ist. Wie sehr helfen die folgenden Maßnahmen, den Heizenergieverbrauch zu senken? Bitte sortieren Sie!

*(1 = größte Einsparung; 4 = kleinste Einsparung)*

1. Das Dach dämmen ***(gestrichen)*** → *– 450 kg $CO_2$-eq / Jahr (KlimAktiv, 2020)*
2. Alle Fenster mit Wärmeschutzverglasung ausstatten ***(gestrichen)***
   → *– 360 kg $CO_2$-eq / Jahr (KlimAktiv, 2020)*
3. Die durchschnittliche Raumtemperatur um 1°C senken
   → *– 300 kg $CO_2$-eq / Jahr (KlimAktiv, 2020)*
4. Keine Fenster auf Kipp lassen, sondern nur Stoßlüften
   → *– 270 kg $CO_2$-eq / Jahr (KlimAktiv, 2020)*

*(Table is continued on next page.)*

**Table1.2 (continiued)**

**EK25**

Bitte sortieren Sie die folgenden Dinge danach, wie sehr sie dem Klima nützen oder schaden (jeweils auf ein Jahr gerechnet).

*(1 = größter Nutzen für das Klima; 4 = größter Schaden für das Klima)*

1. ein Tempolimit auf deutschen Autobahnen von 130 km/h (statt kein Tempolimit)
   → *ca. – 1,9 Mio t $CO_2$-eq / Jahr (Lange, 2020)*
2. Zehn Fußballfelder Wald (statt unbelebter Brachfläche)
   → *ca. – 80 t $CO_2$-eq / Jahr (Bundesinformationszentrum Landwirtschaft [BLE], o. D.), o. D.)*
3. die gesamte Lebensmittelverschwendung in Deutschland (die Treibhausgasemissionen, die auf dem Lebensweg, d. h. für Produktion, Verpackung und Transport all jener Lebensmittel entstehen, die verschwendet werden)
   → *ca. + 48 Mio t $CO_2$-eq / Jahr (Noleppa & Cartsburg, 2015)*
4. alle Braunkohlekraftwerke in Deutschland zusammen
   → *ca. + 150 Mio t $CO_2$-eq / Jahr (Fraunhofer ISE, 2020)*

*Single-Choice-Aufgaben*

**EK13** ***(gestrichen)***

Durch welche der folgenden Handlungen lassen sich am meisten Treibhausgasemissionen einsparen?

- Wenn geheizt wird, Fenster dauernd gekippt lassen, statt sie kurz ganz zu öffnen.
- **Gebäude gut isolieren, vor allem Dächer und Fenster.**
- Gefriergeräte eisfrei halten.
- Die Waschmaschine mit eigenem Warmwasseranschluss versehen.

*Item übernommen aus Gossen et al. (2021)*

**EK14**

Verteilt man die CO2-Emissionen, die bis 2050 maximal noch ausgestoßen werden dürfen, auf alle derzeit lebenden Menschen auf der Erde gleichmäßig, dann hat eine Person ein CO2-Budget von ungefähr 2 Tonnen pro Jahr. Wie viel von diesem Budget verbraucht eine Person für eine Flugreise von Frankfurt am Main nach New York (Hin- und Rückflug)?

- ungefähr 25 % (ein Viertel)
- ungefähr 75 % (drei Viertel)
- ungefähr 100 % (das Ganze)
- **ungefähr 150 % (das Anderthalbfache)**

*In der Economy Class werden ca. 3 t $CO_2$-eq pro Person ausgestoßen (atmosfair gGmbH, 2019)*

**EK15**

Wie viele Treibhausgasemissionen verursacht ein Stück Butter im Vergleich zur selben Menge Margarine?

- etwa halb so viele
- etwa doppelt so viele
- **etwa viermal so viele**
- etwa fünfzehnmal so viele

*Emissionen 250 g Butter: 2.25 kg $CO_2$-eq; 250 g Margarine: 0.56 kg $CO_2$-eq (Reinhardt et al., 2020)*

**EK16**

Ein durchschnittlicher Haushalt in Deutschland verbraucht am meisten Energie für…

- Beleuchtung
- Warmwasser
- **Heizung**
- Elektrogeräte

*Item übernommen aus Geiger et al. (2019)*

*(Table is continued on next page.)*

**Table1.2 (continiued)**

**EK17** ***(gestrichen)***

Wie lange muss eine Windkraftanlage laufen, um die Energie, die für ihren Bau, Betrieb und Rückbau aufgewendet werden, wieder rauszuholen?

- 2 bis 6 Wochen
- **3 bis 7 Monate** *(UBA, 2021)*
- 1 bis 2 Jahre
- 4 bis 5 Jahre

**EK18** ***(gestrichen)***

Bei einem einfachen Flug von Frankfurt am Main nach New York entstehen ungefähr so viele Treibhausgasemissionen, wie wenn eine LED-Leuchte (mit 7 Watt) …

- Ein halbes Jahr durchgängig leuchtet
- Fünf Jahre durchgängig leuchtet
- **60 Jahre durchgängig leuchtet**
- 500 Jahre durchgängig leuchtet

*Emissionen Flug: 1.5 t $CO_2$-eq in der Economy Class (atmosfair gGmbH, 2019); Emissionen Leuchte: 436 g $CO_2$-eq / KWh Strom (KlimAktiv, 2020)*

**EK19** ***(gestrichen)***

Die Treibhausgas-Emissionen, die eine Person am Tag durch einen vollständigen Verzicht auf Tierprodukte in ihrer Ernährung einspart, entsprechen einer Autofahrt von…?

- 700 Metern
- 2,3 Kilometern
- **9,6 Kilometern** → *1.8 kg $CO_2$-eq*
- 18,5 Kilometern

*Emissionen Autofahrt: Allekotte et al. (2021); Emissionen Ernährung: KlimAktiv (2020)*

**EK20** ***(gestrichen)***

Wie lange müsste man vegan leben, um die Emissionen einer Flugreise von Hamburg nach Mallorca und zurück "auszugleichen"?

- ungefähr einen Monat
- ungefähr fünf Monate
- **ungefähr ein Jahr**
- ungefähr zwei Jahre

*Emissionen Flug: 617 kg – 770 kg $CO_2$-eq (atmosfair gGmbH, 2019); Einsparungen durch vegane Ernährung: ca. 680kg $CO_2$-eq im Jahr (KlimAktiv, 2020)*

**EK21**

Beim Kauf von frischen Tomaten im Winter: Welche sollten Sie nehmen, um möglichst wenige Treibhausgase auszustoßen?

- Tomaten aus Deutschland (beheiztes Gewächshaus)
  → *2,9 kg $CO_2$-eq / kg Tomate (Reinhardt et al., 2020)*
- **Tomaten aus Südeuropa/Nordafrika (Freiland)**
  → *0,4 kg $CO_2$-eq / kg Tomate (Reinhardt et al., 2020)*

**EK22**

Bio-Fleisch verursacht weniger Treibhausgasemissionen als konventionelles Fleisch.

- Richtig
- **Falsch** *(Reinhardt et al., 2020)*

**EK23** ***(gestrichen)***

Welches T-Shirt ist das klimafreundlichste?

- **Eins aus Baumwollgewebe** → *4,3 kg $CO_2$-eq (Kirchain et al., 2015)*
- Eins aus Polyestergewebe → *7,1 kg $CO_2$-eq (Kirchain et al., 2015)*
- Eins aus Wolle → *11 kg $CO_2$-eq (Kirchain et al., 2015)*

*(Table is continued on next page.)*

**Table1.2 (continiued)**

**EK24** ***(gestrichen)***

Recycling-Papier ist klimafreundlicher als Papier aus Frischfaser.

- **Richtig** *(Grießhammer et al., 2010)*
- Falsch

**EK26** ***(gestrichen)***

Welches Wasser sollte man trinken, wenn man das Klima schützen möchte?

- Wasser aus 5-Liter-Plastikkanistern
- Wasser aus Plastikflaschen
- Wasser aus Glasflaschen
- **Leitungswasser** *(a tip: tap e.V., 2021)*

*Anmerkung*. Bei den Ordnungsaufgaben entspricht die hier angegebene Ordnung der korrekten Ordnung. Bei den Single-Choice-Aufgaben ist die richtige Lösung durch fetten Schriftsatz gekennzeichnet. Warum Items gestrichen wurden, wird im Laufe des Methodenteils ausgeführt. Grund dafür kann z. B. ein schlechter Fit oder geringe Iteminformation sein.

## References


Allekotte, M., Althaus, H.-J., Bergk, F., Biemann, K., Knörr, W. & Sutter, D. (2021). *Umweltfreundlich mobil! Ein ökologischer Verkehrsartenvergleich für den Personen- und Güterverkehr in Deutschland*. Dessau-Roßlau. Umweltbundesamt (UBA). https://www.umweltbundesamt.de/publikationen/umweltfreundlich-mobil

atmosfair gGmbH. (2019, 12. Juni). *Flug kompensieren - atmosfair: $CO_2$-Fußabdruck meines Flugs berechnen.* atmosfair gGmbH. https://www.atmosfair.de/de/kompensieren/flug/

Bundesamt für Wirtschaft und Ausfuhrkontrolle. (2020, 1. Dezember). *Modul 4 – Energiebezogene Optimierung von Anlagen und Prozessen: Bundesförderung für Energieeffizienz in der Wirtschaft – Zuschuss*. Eschborn. https://www.bafa.de/SharedDocs/Downloads/DE/Energie/eew_modul_4_oap_merkblatt_tma_2020.pdf?__blob=publicationFile&v=2

Bundesinformationszentrum Landwirtschaft (BLE). *Wie viel CO2 binden Wälder?* https://www.landwirtschaft.de/landwirtschaft-verstehen/haetten-sies-gewusst/pflanzenbau/wie-viel-co2-binden-waelder

Climate Watch. (2021). *Historical GHG Emissions.* World Resources Institute. https://www.climatewatchdata.org/ghg-emissions

Deutsche Energie-Agentur GmBH. (2018). *Energiespartipps für die Beleuchtung: Einfach Strom sparen*. Berlin. https://www.dena.de/fileadmin/dena/Publikationen/PDFs/2018/Energiespartipps_fuer_die_Beleuchtung.pdf

Deutsche Umwelthilfe e.V. (2015). *Umweltproblem Coffee to go-Einwegbecher: Die wichtigsten Fakten*. https://www.duh.de/fileadmin/user_upload/download/Projektinformation/Kreislaufwirtschaft/DUH_Coffee_to_go_FactSheet.pdf

Fraunhofer ISE. (2020). *Kohlendioxidemissionen ($CO_2$) von Braunkohlekraftwerken in Deutschland in 2018*. Energy-Charts. https://energy-charts.info/charts/emissions/chart.htm?l=de&c=DE

Geiger, S. M., Geiger, M. & Wilhelm, O. (2019). Environment-specific vs. general knowledge and their role in pro-environmental behavior. *Frontiers in psychology*, *10*, Artikel 718. https://doi.org/10.3389/fpsyg.2019.00718

Gossen, M., Müller, R., Holzhauer, B. & Geiger, S. M. (2021). *"Zukunft? Jugend fragen! 2019"*. Teilbericht (UBA-Texte Nr. 12). Dessau-Roßlau. Umweltbundesamt (UBA). https://www.umweltbundesamt.de/sites/default/files/medien/5750/publikationen/2021-01-21_texte_12-2021_ubs_2019_jugend.pdf

Grießhammer, R., Brommer, E., Gattermann, M., Grether, S., Krüger, M., Teufel, J. & Zimmer, W. (2010). *CO2-Einsparpotenziale für Verbraucher.* Öko-Institut e.V.

Icha, P. (2021). *Entwicklung der spezifischen Kohlendioxid-Emissionen des deutschen Strommix in den Jahren 1990 - 2020* (Climate Change Nr. 45). Dessau-Roßlau. Umweltbundesamt (UBA). https://www.umweltbundesamt.de/publikationen/entwicklung-der-spezifischen-kohlendioxid-7

Initiative Psychologie im Umweltschutz e.V. *Postkarte "Was tust du für den Klimaschutz?"*. https://ipu-ev.de/postkarte/

Kirchain, R., Olivetti, E., Miller, T. R. & Greene, S. (2015). *Sustainable Apparel Materials: An overview of what we know and what could be done about the impact of four major apparel materials: Cotton, Polyester, Leather, & Rubber* (1) [, Massachusetts Institute of Technology, Cambridge, MA]. CrossRef.

KlimAktiv gemeinnützige Gesellschaft zur Förderung des Klimaschutzes mbH. (2020). *CO2-Rechner des Umweltbundesamtes*. https://uba.co2-rechner.de/de_DE/

Lange, M. (2020). *Klimaschutz durch Tempolimit: Wirkung eines generellen Tempolimits auf Bundesautobahnen auf die Treibhausgasemissionen* (UBA-Texte 38/2020). Dessau-Roßlau. Umweltbundesamt (UBA). https://www.umweltbundesamt.de/publikationen/klimaschutz-durch-tempolimit

Noleppa, S. & Cartsburg, M. (2015). *Das große Wegschmeißen: Vom Acker bis zum Verbraucher: Ausmaß und Umwelteffekte der Lebensmittelverschwendung in Deutschland.* WWF Deutschland. https://www.wwf.de/fileadmin/fm-wwf/Publikationen-PDF/WWF_Studie_Das_grosse_Wegschmeissen.pdf

Reinhardt, G., Gärtner, S. & Wagner, T. (2020). *Ökologische Fußabdrücke von Lebensmitteln und Gerichten in Deutschland*. Heidelberg. ifeu Institut für Energie- und Umweltforschung. https://www.ifeu.de/projekt/oekologischer-fussabdruck-von-lebensmitteln-und-gerichten-in-deutschland/

a tip: tap e.V. (2021). *Fünf Gründe für Leitungswasser*. https://atiptap.org/wasserwissen/5-gruende-fuer-leitungswasser/

Umweltbundesamt. (2021). *Windenergie an Land*. https://www.umweltbundesamt.de/themen/klima-energie/erneuerbare-energien/windenergie-an-land#flaeche

Verbraucherzentrale Nordrhein-Westfalen e.V. (Hrsg.). (2017). *Duschrechner: Dreh auf und spar!* Verbraucherzentrale Nordrhein-Westfalen e.V. http://projekte.meine-verbraucherzentrale.de/DE-NW/duschrechner.html
