# Supplementary material for: Better climate action through the right knowledge? Development and validation of an item-response-theory scale measuring climate effectiveness knowledge
Source: Front Psychol. 2024 Nov 14;15:1347407. doi: 10.3389/fpsyg.2024.1347407 (PMC11605514; doi:10.3389/fpsyg.2024.1347407)
Supplement: Supplementary file 2 [file Table_2.pdf]

Appendix A2: High-Impact Pro-Environmental Behavior Scale

Instruction:
„Now I would like to ask you for a few more details about your behavior. Please indicate how often you perform the following actions. Please mark "no information" if a question does not apply to your current life situation. (For example, you cannot give information about your compensation for air travel if you never fly.)"

Table A2.1
Items of the high-impact PEB scale, Likert-Items

Short form	Item	Response options	coding
PEB_ways	For my everyday trips (to school / university / work, to the grocery store) I use the bicycle, public transport or walk.	Never	0
		Rarely	1
		Occasionally	2
		Often	3
		Very often / always	4
		No statement	-
PEB_comp	I compensate for air travel by donating to reforestation projects, for example.	Never	0
		Rarely	1
		Occasionally	2
		Often	3
		Very often / always	4
		No statement	-
PEB_led	In my household, I invest in energy-saving appliances (e.g. refrigerator, washing machine, LED lighting).	Never	0
		Rarely	1
		Occasionally	2
		Often	3
		Very often / always	4
		No statement	-
PEB_consume	When I buy something new, I check carefully (e.g., on the Internet) whether it is durable and resource-saving.	Never	0
		Rarely	1
		Occasionally	2
		Often	3
		Very often / always	4
		No statement	-
PEB_donation	I donate money to environmental organizations.	Never	0
		Rarely	1
		Occasionally	2
		Often	3
		Very often / always	4
		No statement	-
PEB_demo	I go to political demonstrations or protests demanding for environmental or climate protection.	Never	0
		Rarely	1
		Occasionally	2
		Often	3
		Very often / always	4
		No statement	-
PEB_season	I buy fruits and vegetables in season.	Never	0
		Rarely	1
		Occasionally	2
		Often	3
		Very often / always	4
		No statement	-

PEB_wash	I use a tumble dryer.	Never<br>Rarely<br>Occasionally<br>Often<br>Very often / always<br>No statement	4<br>3<br>2<br>1<br>0<br>-
PEB_car1	For trips to the surrounding area (up to 30 km) I use the car.	Never<br>Rarely<br>Occasionally<br>Often<br>Very often / always<br>No statement	4<br>3<br>2<br>1<br>0<br>-
PEB_heat	In my apartment in winter it is warm enough that you are not cold without a sweater.	Never<br>Rarely<br>Occasionally<br>Often<br>Very often / always<br>No statement	4<br>3<br>2<br>1<br>0<br>-

Table A2.2

Items of the high-impact PEB scale, dichotomous items

Short form	Item	Response options	coding
PEB_ship	I have taken a multi-day ship trip/cruise in the last 12 months.	No<br>Yes	Item not included because it did not show variance
PEB_solar	I have purchased a solar system for energy or heat production.	No<br>Yes	0<br>4
PEB_invest	I have invested in a climate-friendly fund or bank.	No<br>Yes	0<br>4

Table A2.3

Items of the high-impact PEB scale, items with a different response format

Short form	Item	Response options	coding
PEB_flight	How many hours have you flown in the past 12 months? [If you have not flown, please enter "0"]	___ flight hours	0 hours: 4<br>1 - 3 hours: 3<br>4 - 5 hours: 2<br>6 - 10 hours: 1<br>> 10 hours: 0
PEB_car2	Do you own a fuel-efficient car (less than 6 liters of fuel per 100 km) or an electric car?	Yes, and I primarily / only use this car.<br>Yes, but only as a second car.<br>No, but I own one or more cars.<br>No, I do not own a car at all.	3<br>1<br>0<br>4

PEB_nutri:	Which term best describes your diet?	Vegan (complete abstention from animal products)	4
		Vegetarian (abstention from meat and fish)	3
		Little meat (approx. twice a week meat / average 50 g meat per day)	2
		Mixed diet (approx. one portion of meat per day / average 165 g meat per day)	1
		Meat-based diet (more than one portion of meat per day / average 290 g meat per day)	0
PEB_green	Do you currently purchase green electricity?	Yes, and my electricity provider is a certified "green electricity provider" (label "Grüner Strom" or "OK Power")	4
		Yes, but my electricity provider is not certified with one of the above labels or I do not know if it is certified.	2
		No.	0
		Electricity is part of my rent / I have no control over my electricity provider.	-
		I don't know what kind of electricity I get.	0
